# Supplementary material for: Dynamically crossing diabolic points while encircling exceptional curves: A programmable symmetric-asymmetric multimode switch
Source: Nat Commun. 2023 Apr 12;14:2076. doi: 10.1038/s41467-023-37275-5 (PMC10097868; doi:10.1038/s41467-023-37275-5)
Supplement: Supplementary file 1 — Supplementary Information [file 41467_2023_37275_MOESM1_ESM.pdf]

Supplementary Information:
Dynamically crossing diabolic points while encircling exceptional curves: A programmable symmetric-asymmetric multimode switch

Ievgen I. Arkhipov *et al.*

SUPPLEMENTARY NOTE 1. DYNAMICALLY CROSSING DIABOLIC POINTS WHILE ENCIRCLING AN EXCEPTIONAL CURVE IN PASSIVE $\mathcal{PT}$-SYMMETRIC FOUR-MODE SYSTEM

In this section we discuss the symmetric-asymmetric mode switching mechanism in the *passive* $\mathcal{PT}$-symmetric system consisting of four coupled cavities or waveguides. The schematic realization for such a system is identical to that in Fig. 1 in the main text, with the exception that the gain now can be smaller than losses. We set the mode coupling $k = 0.5$ (which also sets the system units), to ensure the full encirclement of EPs for the same modulation functions of the system parameters as those in Eq. (7) in the main text. The corresponding perturbed NHH reads

$$\hat{H}(\delta) = \begin{pmatrix} iA(t)+\delta(t) & g(t) & 0.5 & 0 \\ g(t) & iA(t) & 0 & 0.5 \\ 0.5 & 0 & -i\Delta(t) & g(t) \\ 0 & 0.5 & g(t) & -i\Delta(t)-\delta(t) \end{pmatrix}, \tag{S1}$$

where the modulated gain is $A(t) = A[1+\cos(\omega t+\phi_0)]$, such that $0 < A < \Delta$. Moreover, $\Delta(t)$ and $g(t)$ are the time-dependent dissipation and mode-coupling strength, respectively, and $\delta(t)$ is perturbation given in Eq. (7) in the main text. And ω (ϕ_0) is the winding frequency (initial phase) of the modulation.

The spectrum of the NHH in Eq. (S1) with zero gain $A = 0$ and mode coupling $g > 0$ is shown in Fig. 1. We confirm that for zero gain $A = 0$, the mode-switching mechanism remains the same as for the system with balanced gain and loss, as discussed in the main text. We plot the fidelity $|\langle\psi_k|\psi(t)\rangle|^2$ of the NHH eigenstates (ψ_k) at times t and the time-evolving state ($\psi(t)$) in Fig. 2. The symbol $\langle\cdot|\cdot\rangle$ denotes the Hilbert inner product of two states. As one can see, the state evolution is reminiscent to that in Fig. 4 in the main text, though it has some additional peculiarities. For instance, one can observe some extra mode switching at times $t = T/2, 3T/2$ when the system does not cross the DC, e.g., a seemingly extra swap between states ψ_2 and ψ_3 at time $T/2 < t < T$ in panel (c) in Fig. 2. This extra state flip is nothing else but just a reordering of the Riemann sheets ($E_2 \to E_3$ and $E_3 \to E_2$), when the two separated Riemann surface pairs start intersecting each other while moving along the vertical axis in Fig. 1, which is caused by the mode coupling modulation $g(t)$. Moreover, at larger times $t \approx 2T$, instabilities can appear [see panels (a)-(b), and (g)-(h) in Fig. 2]. However, the mode switching mechanism (as presented in Table 1 in the main text) remains unchanged for the full double period $2T$. Also, with decreasing gain a problem with the fine tuning in the winding speed may emerge. Nevertheless, by applying the gain, such that $A < \Delta$, one can remove the arising instabilities as shown in Fig. 3, as well as other problems related the mentioned parameter constraints.

We have, thus, demonstrated that our findings, presented in the main text, remain valid also for passive $\mathcal{PT}$-symmetric systems which might be useful for classical low-power and even quantum optical applications.

SUPPLEMENTARY NOTE 2. DYNAMICALLY CROSSING DIABOLIC POINTS WHILE ENCIRCLING AN EXCEPTIONAL CURVE IN AN EIGHT-MODE $\mathcal{PT}$-SYMMETRIC SYSTEM

Here we further extend out results to an eight-mode $\mathcal{PT}$-symmetric system. Following the same approach we described in the main text, one can construct an eight-mode NHH out of combination of a $\mathcal{PT}$-symmetric dimer and some 4×4 Hermitian matrix with DPs. Namely, by taking

$$M_1 = \begin{pmatrix} i\Delta & k \\ k & -i\Delta \end{pmatrix}, \quad M_2 = \begin{pmatrix} 0 & g & 0 & 0 \\ g & 0 & l & 0 \\ 0 & l & 0 & g \\ 0 & 0 & g & 0 \end{pmatrix}, \tag{S2}$$

one can construct a new matrix H as follows:

$$H = M_1 \otimes I_4 + I_2 \otimes M_2, \tag{S3}$$

where I_2 (I_4) is the 2×2 (4×4) identity matrix.

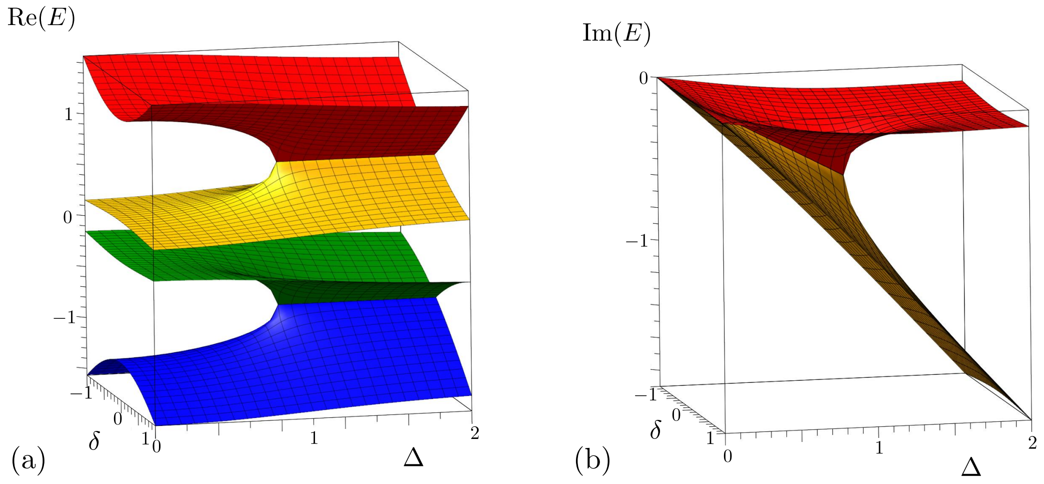

FIG. 1. Spectrum of the passive $\mathcal{PT}$-symmetric four-mode system. (a) Real and (b) imaginary parts of the spectrum of the NHH as a function of dissipation strength Δ and the perturbation δ, according to Eq. (S1). The system parameters are: $A = 0$ and $g = 0.7$.

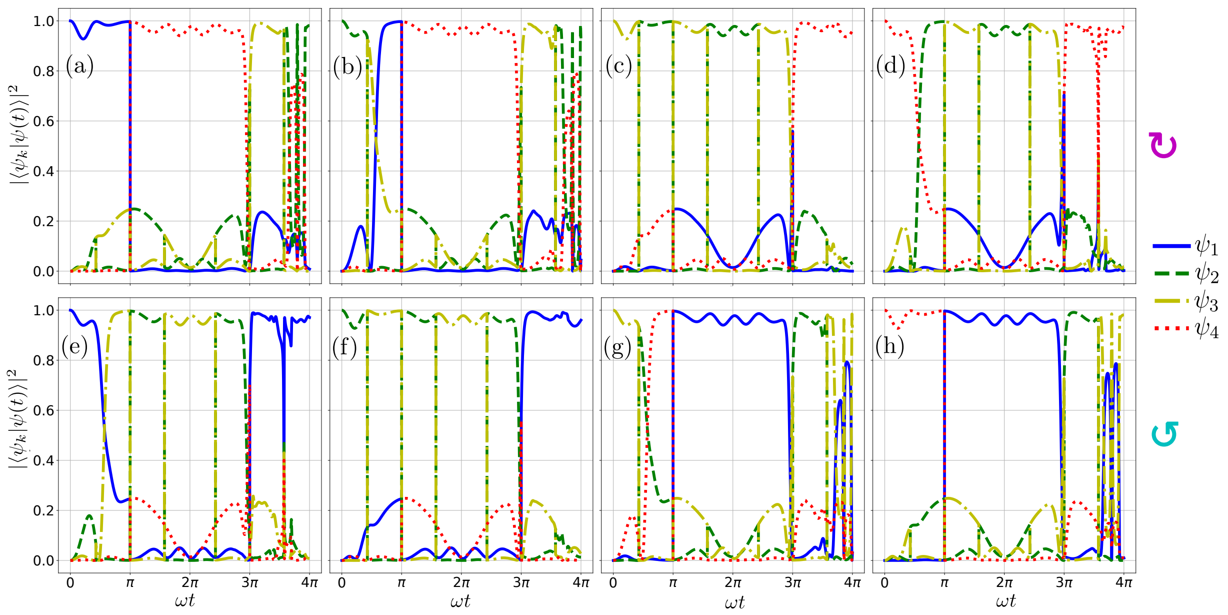

FIG. 2. A programmable passive $\mathcal{PT}$-symmetric four-mode switch, governed by the NHH in Eq. (S1), as indicated by the fidelity of the NHH eigenstates ψ_k at time t, and the time-evolving state $\psi(t)$ during a double period $2T$. The initial eigenmodes ψ_k, $k = 1, \ldots, 4$, are located in the exact $\mathcal{PT}$-phase. Panels (a)-(d) and panels (e)-(h) are respectively obtained for clockwise and counterclockwise encircling directions. The system parameters are: $\phi_0 = \pi$, $\omega t = \pi t/22.2$, and $g = 0.7$.

The matrix H may correspond to the $\mathcal{PT}$-symmetric NHH, written in the mode representation and describing an

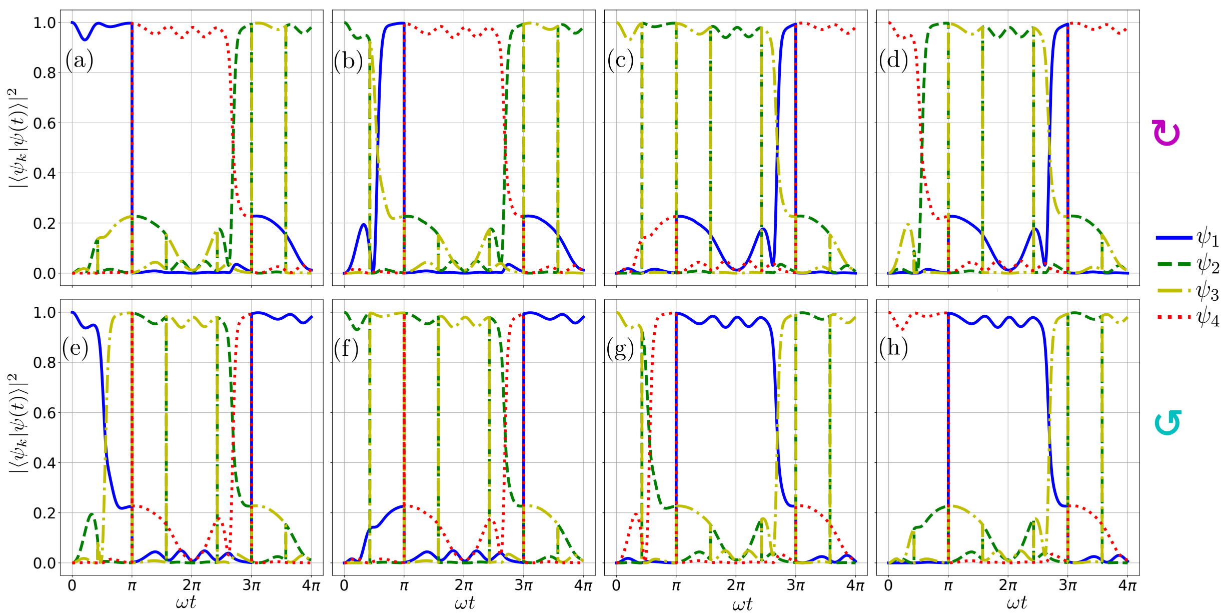

FIG. 3. A programmable passive $\mathcal{PT}$-symmetric four-mode switch, similar to that in Fig. 2, but with the nonzero gain $A = 0.1$, according to Eq. (S1). The remaining system parameters are the same as in Fig. 2.

eight-mode bosonic system. In analogy with Eq. (6) in the main text, we perturb this NHH in the following way:

$$\hat{H}(\delta) = \begin{pmatrix} i\Delta+\delta & g & 0 & 0 & k & 0 & 0 & 0 \\ g & i\Delta+\delta & l & 0 & 0 & k & 0 & 0 \\ 0 & l & i\Delta & g & 0 & 0 & k & 0 \\ 0 & 0 & g & i\Delta & 0 & 0 & 0 & k \\ k & 0 & 0 & 0 & -i\Delta & g & 0 & 0 \\ 0 & k & 0 & 0 & g & -i\Delta & l & 0 \\ 0 & 0 & k & 0 & 0 & l & -i\Delta-\delta & g \\ 0 & 0 & 0 & k & 0 & 0 & g & -i\Delta-\delta \end{pmatrix}. \tag{S4}$$

The schematic representation of the eight-mode system governed by the NHH in Eq. (S4) is shown in Fig. 4. In what follows, we set $k = 1$ (establishing system units) and modulate the system parameters as:

$$\begin{aligned} \Delta(t) &= 1+\cos(\omega t+\phi_0), \\ g(t) &= \cos^2(\omega t/2), \\ l(t) &= \cos^2(\omega t/2), \\ \delta(t) &= \sin(\omega t+\phi_0). \end{aligned} \tag{S5}$$

A typical spectrum of such a system is illustrated in Fig. 5(a). This spectrum consists of 4 separated pairs of Riemann sheets. By appropriately modulating either the mode couplings g or l, one can realize various four-mode switching combinations in analogy with those in the main text. That is, modulating only coupling $g(t)$ and leaving $l = \text{const}$ allows one to implement a symmetric-asymmetric switching between four states $\psi_2 \leftrightarrow \psi_4 \leftrightarrow \psi_5 \leftrightarrow \psi_7$ [see Figs. 5(a)-(c)]. The modulation of only $l(t)$ with $g = \text{const}$ ensures the swapping between two pairs of four-mode states: $\psi_1 \leftrightarrow \psi_2 \leftrightarrow \psi_3 \leftrightarrow \psi_4$ and $\psi_5 \leftrightarrow \psi_6 \leftrightarrow \psi_7 \leftrightarrow \psi_8$ [see Fig. 5(d)-(f)]. The switching order in all these three four-mode combinations is similar to that in the main text. For instance, the state swapping between the states $\psi_2 \leftrightarrow \psi_4 \leftrightarrow \psi_5 \leftrightarrow \psi_7$ is identical to the state switching order between the states $\psi_1 \leftrightarrow \psi_2 \leftrightarrow \psi_3 \leftrightarrow \psi_4$ in the main text. The same applies to other two four-mode compositions. We additionally confirm these results by plotting the fidelity between the initial eigenstates $\psi_k(0)$ at time $t = 0$ and the time-evolving state $\psi(t)$ in Figs. 6 and 7. As such,

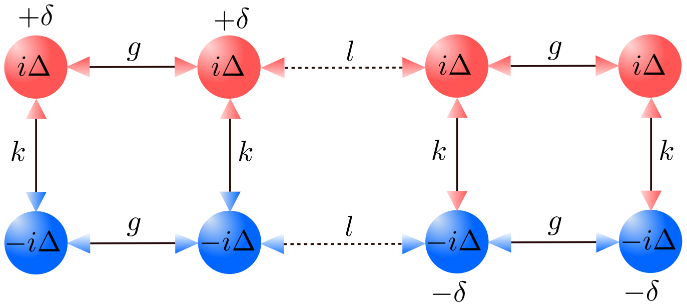

FIG. 4. Schematic representation of an eight-mode $\mathcal{PT}$-symmetric non-Hermitian Hamiltonian $\hat{H}$, given in Eq. (S1). The red (blue) balls represent cavities with gain (loss) rate $i\Delta$ ($-i\Delta$). Various mode couplings are depicted by double arrows. Four cavities are coherently perturbed by the frequency detuning $\pm\delta$.

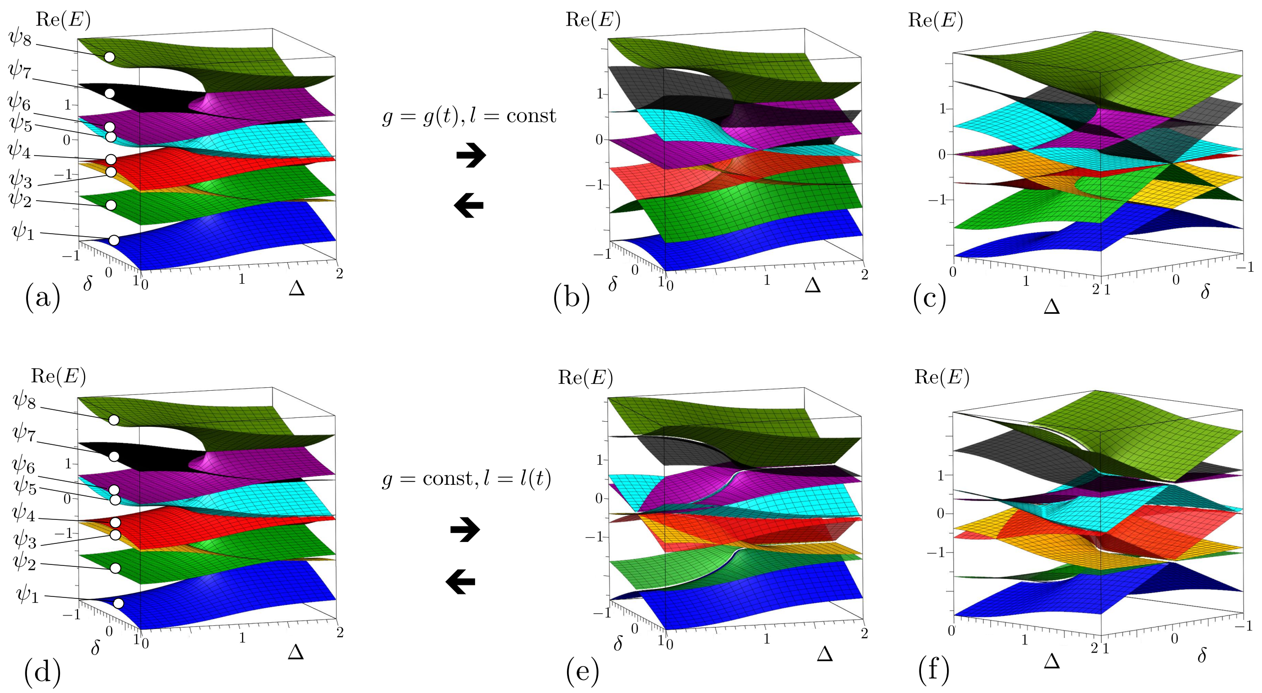

FIG. 5. Real spectrum of the eight-mode $\mathcal{PT}$-symmetric system in the parameter space (Δ, δ) depending on the mode-coupling modulation. (a)-(c) Shows the cases for the modulated coupling $g(t)$ with constant coupling $l = 1$, and (d)-(f) for the modulated coupling $l(t)$ with constant coupling $g = 1$. Panels (a) and (d) correspond to the initial condition when $g = l = 1$; panels (b)-(c) correspond to the case when $g = 0$, and panels (e)-(f) to the case when $l = 0$, respectively. It can be seen that, depending on the choice of the mode coupling modulation, various pairs of Riemann surfaces may form DCs, and, thus, various state swapping combinations can be realized on demand.

by alternating the modulation between mode couplings g and l one can implement a symmetric-asymmetric mode switching for all the eight modes in the system.

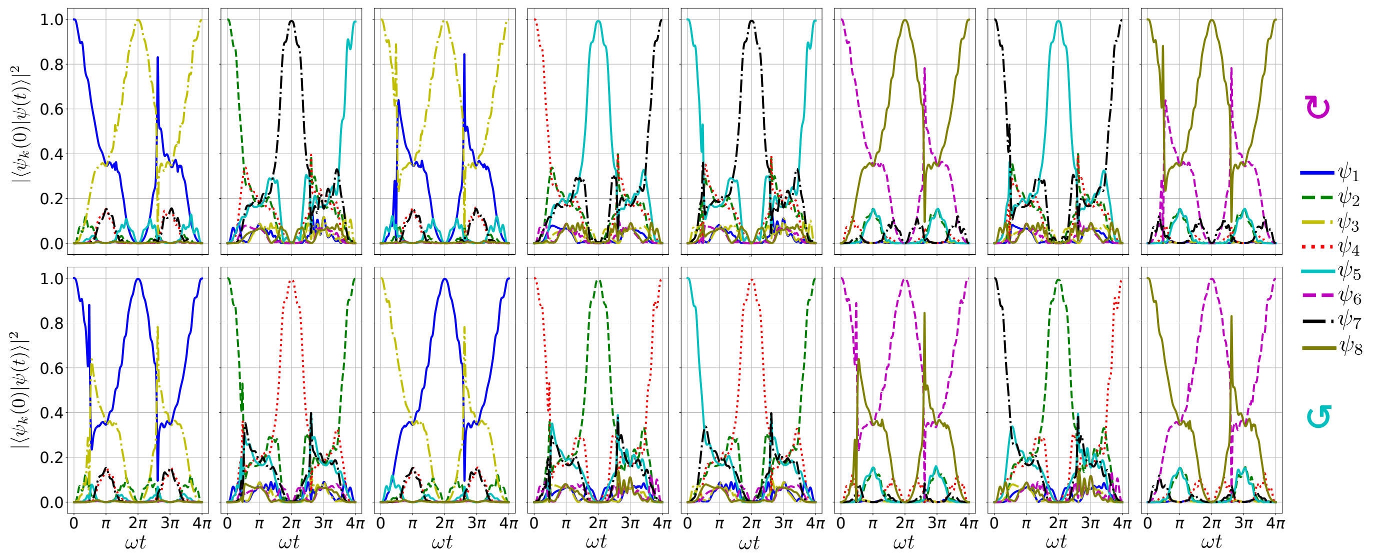

FIG. 6. A programmable eight-mode switch by dynamically encircling an exceptional curve, EC, while crossing a diabolic curve, DC, as indicated by the fidelity of the initial states $\psi_k(0)$ at $t = 0$, and the time-evolving $\psi(t)$ state during a double period $2T$ with mode coupling modulation $g(t)$. The system parameters are: $l = 1$, $\omega t = \pi t/55$.

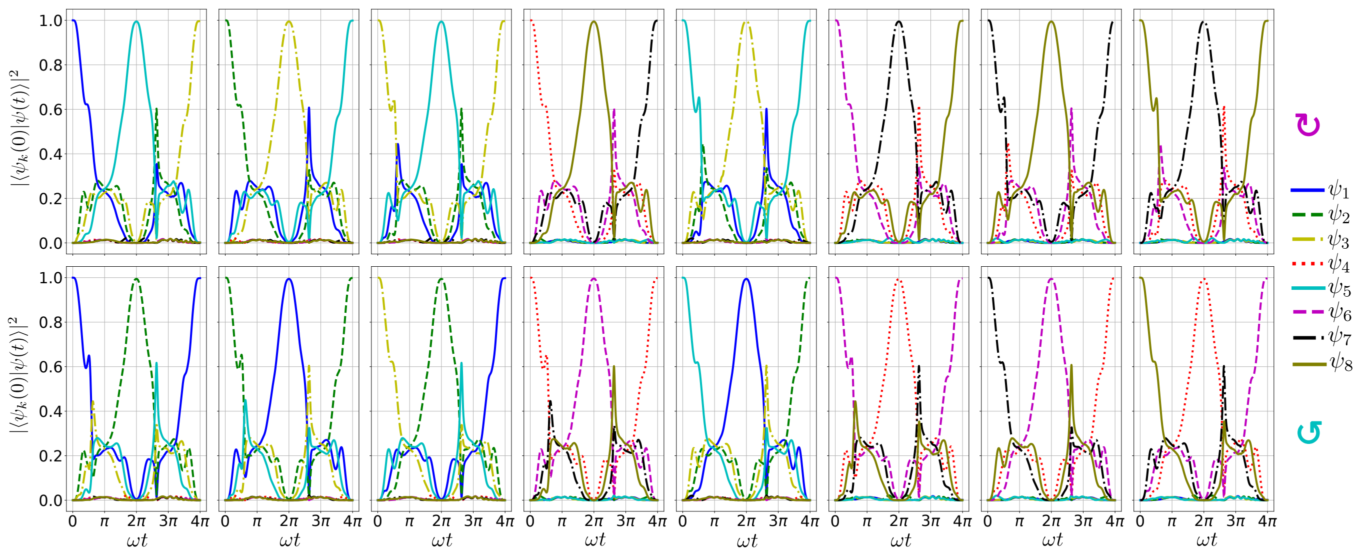

FIG. 7. A programmable eight-mode switch by dynamically encircling an exceptional curve, EC, while crossing a diabolic curve, DC, as indicated by the fidelity of the initial states $\psi_k(0)$ at $t = 0$, and the time-evolving $\psi(t)$ state during a double period $2T$ with mode coupling modulation $l(t)$. The system parameters are: $g = 1$, $\omega t = 2\pi t/55$.
